# Neuro-ophthalmic complications of tuberculosis and its treatment: a systematic review and meta-analysis

**DOI:** 10.3389/fopht.2026.1818640

**Published:** 2026-05-29

**Authors:** Ahmed A. Alessa, Ahmed Zuhier Awan, Abdulkarim B. Almutairi, Ibrahim Ghassan Ajabnoor, Mohammed Marouf M. Jan, Ahmed Y. Azzam, Nooran Badeeb

**Affiliations:** 1 Faculty of Medicine, King Abdulaziz University, Jeddah, Saudi Arabia; 2 School of Medicine, Trinity College Dublin, Dublin, Ireland; 3 Department of Clinical Research and Clinical Artificial Intelligence, ASIDE Healthcare, Lewes, DE, United States; 4 Division of Global Health and Public Health, School of Nursing, Midwifery and Public Health, University of Suffolk, Suffolk, United Kingdom; 5 Ophthalmology Surgery, Division of Ophthalmology, College of Medicine, University of Jeddah, Jeddah, Saudi Arabia; 6 Department of Ophthalmology, King Fahad Armed Forces Hospital, Jeddah, Saudi Arabia

**Keywords:** ethambutol, neuro-ophthalmology, optic neuropathy, tuberculosis, tuberculous meningitis, visual evoked potentials

## Abstract

**Introduction:**

Tuberculosis (TB) and its treatment have been associated with significant neuro-ophthalmic morbidity; however, the magnitude and determinants of these complications remain incompletely characterized. This systematic review and meta-analysis aimed to evaluate the incidence and risk factors of ethambutol optic neuropathy (EON), neuro-ophthalmic manifestations of tuberculous meningitis (TBM), and prognostic biomarkers for visual outcomes.

**Methods:**

Following PRISMA 2020 guidelines, we searched PubMed, Scopus, Embase, Web of Science, Cochrane Library, and Google Scholar up to the 30th of September 2025. Studies reporting EON incidence, TBM neuro-ophthalmic complications, or subclinical neurotoxicity biomarkers were included. Random-effects meta-analysis with generalized linear mixed models was performed.

**Results:**

Twenty-two studies (N = 260,430) were included. Pooled EON incidence was 1.54% (95% CI: 0.81-2.49%, I²=98.2%). Renal impairment (OR 3.73, 95% CI: 1.78-7.83) and hypertension (OR 2.37, 95% CI: 1.46-3.84) were significant risk factors. TBM neuro-ophthalmic manifestations included cranial nerve III palsy (17.4%), papilledema (12.5%), and optic atrophy (16.7%), with pediatric patients demonstrated significantly higher hydrocephalus rates (72.5% vs 13.0%, RR 5.56). Visual evoked potential (VEP) demonstrated better detection of subclinical changes over optical coherence tomography (OCT) (Hedges’ g difference: 0.686, P-value = 0.001). Visual recovery occurred in 52.4% of clinical EON cases. Factors associated with improved recovery included younger age (MD= -3.8 years, P-value= 0.095) and earlier ethambutol discontinuation.

**Conclusions:**

TB-related neuro-ophthalmic complications represent significant morbidity with identifiable risk factors. Visual evoked potentials offer superior subclinical detection and early intervention improves visual outcomes. Screening protocols targeting high-risk populations are recommended.

**Systematic review registration:**

https://www.crd.york.ac.uk/PROSPERO/, identifier CRD420251141453.

## 1 Introduction

Tuberculosis remains a leading infectious cause of both morbidity and mortality around the world, with around 10.6 million new cases diagnosed annually. While pulmonary manifestations predominate, extrapulmonary involvement, including neuro-ophthalmic complications, represents a significant source of disability that has received comparatively less attention in the literature. The visual system may be affected through multiple mechanisms, including direct infection of the ocular and neural structures, inflammatory responses to mycobacterial antigens, and iatrogenic toxicity from anti-tuberculous medications (1–3).

Ethambutol, one of the first-line agents in standard anti-tuberculous regimens, has been associated with optic neuropathy since its introduction in the 1960s. Ethambutol optic neuropathy (EON) manifests as bilateral visual loss with characteristic red-green dyschromatopsia and central or cecocentral scotomas, resulting from toxicity to ganglion cells and the retinal nerve fiber layer.

Despite widespread recognition of this complication, the reported incidence varies significantly across studies, ranging from less than one per cent to over three per cent, with considerable uncertainty regarding modifiable risk factors and optimal screening strategies (4–6).

Tuberculous meningitis (TBM) represents a major cause of neuro-ophthalmic morbidity, especially in endemic regions. The inflammatory response within the central nervous system leads to cranial neuropathies, papilledema from elevated intracranial pressure, and optic atrophy from direct involvement or secondary complications. Pediatric populations appear differentially affected by TBM-related visual sequelae; however, the magnitude of age-related differences has not been systematically quantified (7, 8).

Recent advances in diagnostic technology have demonstrated the possible utility of subclinical biomarkers for early detection of optic nerve dysfunction before irreversible visual loss occurs. Visual evoked potentials (VEP) and optical coherence tomography (OCT) have emerged as promising monitoring tools; however, their comparative sensitivity for subclinical neurotoxicity detection remains uncertain (9–11).

Despite the accumulating evidence on TB-related neuro-ophthalmic complications, no structured meta-analysis has evaluated the pooled incidence of EON, quantified risk factors, and compared adult versus pediatric TBM neuro- ophthalmic manifestations, or assessed the diagnostic performance of subclinical biomarkers. To address these evidence gaps, we aimed to conduct a systematic review and a meta-analysis to evaluate the incidence and risk factors of EON, characterize neuro-ophthalmic manifestations of TBM across age groups, and compare the sensitivity of VEP versus OCT for subclinical detection.

## 2 Methods

### 2.1 Search strategy and study selection

This systematic review and meta-analysis were conducted according to the Preferred Reporting Items for Systematic Reviews and Meta-Analyses (PRISMA) 2020 guidelines (12) and PROSPERO registered (CRD420251141453). We performed a detailed literature search in PubMed, Embase, Scopus, Web of Science, Cochrane Central Register of Controlled Trials, and Google Scholar from database inception up to the 30th of September 2025, focusing on English-language-based studies only. The search strategy utilized a combination of Medical Subject Headings (MeSH) terms and free-text keywords designed to capture all relevant studies investigating neuro-ophthalmic complications of tuberculosis and its treatment. The search string included terms related to tuberculosis, ethambutol, optic neuropathy, TBM, VEPs, and OCT combined with appropriate Boolean operators. Reference lists of all included studies and relevant systematic reviews were manually screened to identify additional eligible studies not captured by electronic searches.

We first conducted title and abstract screening according to our eligibility criteria, with discrepancies resolved through discussion or consultation with a third reviewer when necessary. Studies were included if they met the following criteria: observational studies [prospective cohorts, retrospective cohorts (or case-control studies)] reporting EON incidence, TBM neuro-ophthalmic manifestations, or subclinical neurotoxicity biomarkers; clear definition of outcomes with specification of diagnostic criteria; and reporting of quantitative data sufficient for meta-analysis. Studies were excluded if they were case reports or case series with fewer than ten patients, lacked adequate outcome definitions, or provided insufficient data for quantitative synthesis despite contact attempts with authors.

### 2.2 Data extraction and quality assessment

Data extraction was performed to extract the following information: study characteristics (first author, publication year, country, study design, sample size, follow-up duration); patient demographics and baseline characteristics (age, gender distribution, TB type, comorbidities); intervention or exposure details (ethambutol dosing, treatment duration), and all reported outcomes with raw data, including event counts and denominators for proportional outcomes or means and standard deviations for continuous outcomes. When studies reported outcomes as medians with interquartile ranges rather than means with standard deviations, we converted these using established statistical methods. Authors were contacted via email for missing data or clarification when necessary.

Risk of bias in included studies was assessed using the Newcastle-Ottawa Scale (NOS) for observational studies. Domains assessed included selection of study groups, comparability of groups, and ascertainment of outcomes. Studies scoring seven or more out of nine were considered low risk of bias, four to six as moderate risk, and less than four as high risk.

### 2.3 Outcomes evaluation and assessment

The primary outcome was the pooled incidence of EON among patients receiving ethambutol- containing regimens. Secondary outcomes included risk factors for EON development, neuro-ophthalmic manifestations of TBM (cranial nerve palsies, papilledema, optic atrophy, hydrocephalus, mortality), comparative sensitivity of VEP versus OCT for subclinical detection, and visual recovery rates following EON diagnosis.

### 2.4 Statistical analysis

All statistical analyses were performed using random-effects meta-analysis models based on the DerSimonian-Laird method to account for anticipated heterogeneity across studies. For proportional outcomes, we utilized generalized linear mixed models (GLMM) with a logit transformation to calculate pooled proportions with corresponding 95% confidence intervals (CI) (13). For continuous outcomes, we calculated weighted mean differences or standardized mean differences (Hedges’ g) with 95% CI. Statistical heterogeneity was quantified using the I² statistic, with values exceeding 50% considered significant heterogeneity. Subgroup analyses were conducted, stratifying studies by study design, population type, and publication era. Publication bias was assessed through visual inspection of funnel plots and quantified using Egger’s regression test and Begg’s rank correlation test. Certainty of evidence was evaluated using the Grading of Recommendations Assessment, Development and Evaluation (GRADE) framework (14, 15). All analyses were conducted using RStudio statistical software version 2025.09 with R version 4.4.2.

## 3 Results

### 3.1 Study selection and characteristics

**Figure 1:**
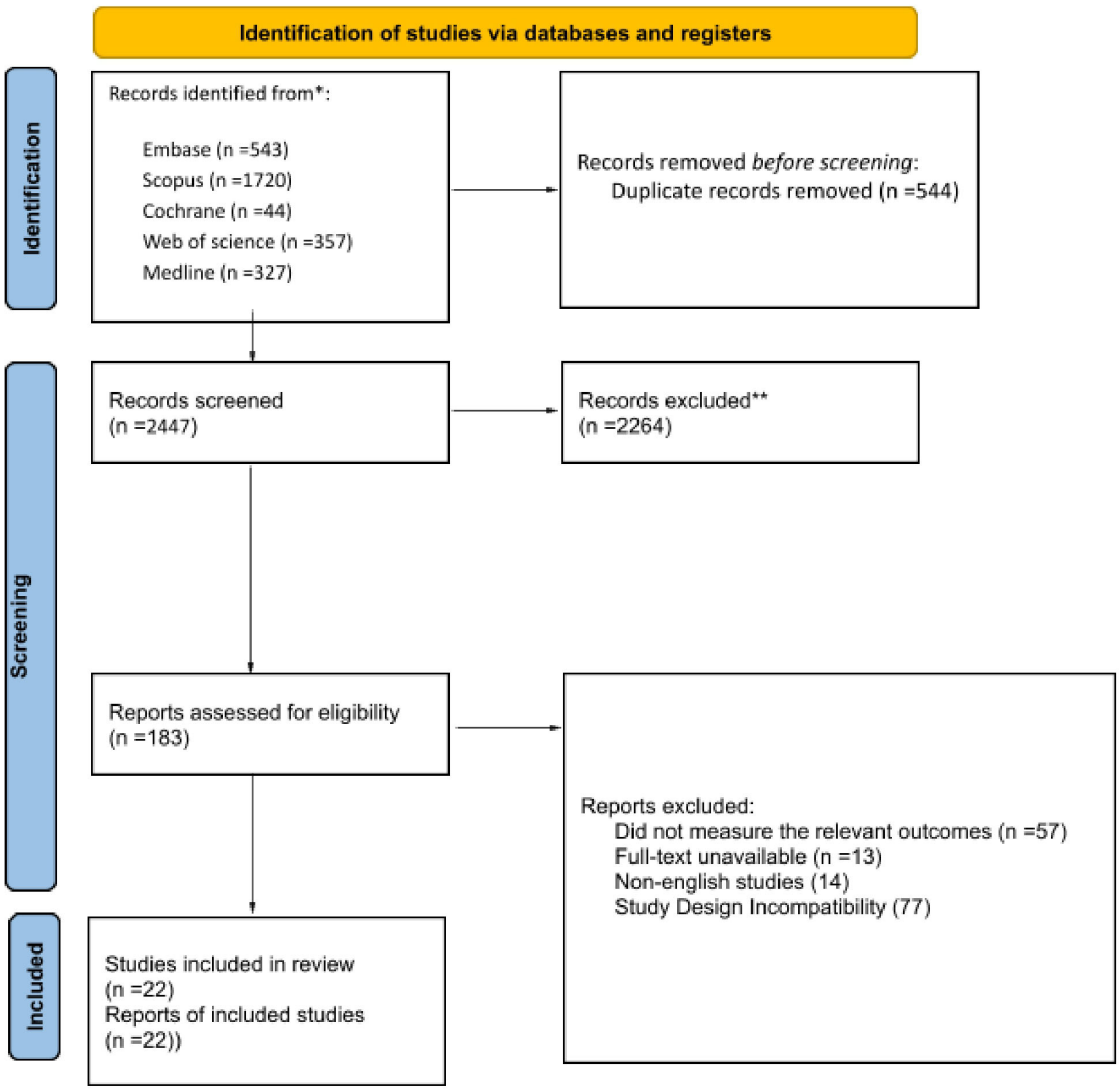
PRISMA flow diagram of study selection.

The literature search identified 2991 records from electronic databases (**Figure 1**). After removing duplicates and records marked as ineligible by automation tools, 2447 unique records underwent title and abstract screening. Following exclusion of irrelevant records, 183 reports were retrieved for full-text assessment. After full-text review, 161 reports were excluded, resulting in 22 studies included in our study for quantitative synthesis. Most studies were predominantly observational, with VEP data derived from retrospective and prospective cohorts conducted in hospitals or specialized eye centers. Four studies were either population-based or surveillance studies with VEP data derived from national databases. Across In studies, VEP was performed as part of a targeted ophthalmic evaluation in patients presenting with visual symptoms or at high risk of EON.

Study characteristics, patient demographics, and clinical details are presented in **Table 1**. The included studies were published between 1985 and 2025, enrolling a total of 260,430 patients from 11 countries across Asia, Europe, Australia, and multinational collaborations. Study designs included population-based cohorts, retrospective cohorts, prospective cohorts, and case-control studies. The mean age of patients ranged from pediatric populations aged from less than 12 months to 20 years, The adult population ranged from 20- to 70-year-olds across studies. 150,317 patients are male, ranging from 52% to 74%, between the populations of the included studies. TB types included pulmonary TB, extrapulmonary TB, TBM, and ocular TB. Follow-up duration ranged from two months to 30 months when reported.

**Table 1:** Baseline demographics and study characteristics of included studies.

Study	Country	Design	Setting	Period	Total N	Pediatric N	Age (years)	Male (%)	TB type	Follow-up	Primary focus
Kim et al., 2025 (30)	Korea	PBC	National database	2015– 2021	119,636	0	60.2 ± 19	57.9	Pulmonary	–	EON screening
Deepa et al., 2024 (31)	India	CS	Tertiary hospital	2019– 2020	143	8	>50: 46%	63.6	Pulm/Ext rapulm	–	Ocular TB
Fei et al., 2024 (32)	China	RC	Public health center	2013– 2022	250	3	26.5‡	61.6	TBM	5.5 mo	TBM neuro-ophth
Kim et al., 2024 (33)	Korea	PBC	National database	2015– 2021	117,309	0	60.1 ± 19	58.1	Pulmonary	–	EON incidence
Chaitanu wong et al., 2023 (21)	Thailand	RC	Tertiary hospital	2012– 2019	4,141	–	43.7	–	Pulm/Ext rapulm	–	EON incidence
Ambika et al., 2022 (34)	India	RC	Tertiary eye center	2017– 2019	128	–	50.6 ± 15	57.0	Pulm/Ext rapulm	6 mo	EON features
Fattore et al., 2022 (abstract) (35)	Australia	RC	Single center	–	161	–	–	–	Systemic	–	EON incidence
Shirley et al., 2020 (36)	UK	PS	National surveilla nce	2016– 2017	48	3	49 ± 18	58.0	Ocular	12 mo	Ocular TB surv
Jin et al., 2019 (28)	Korea	RC	Tertiary hospital	2014– 2016	84	0	53.6 ± 17	60.0	Pulm/Ext rapulm	6 mo	Subclinical EON
Li et al., 2019 (23)	China	RC	TB hospital	2014– 2017	486	3	35.2 ± 17	52.3	TBM	2 mo	TBM CN palsies
Kanaujia et al., 2018 (37)	India	PC	Tertiary hospital	–	23	0	42.6 ± 14	65.0	Renal	3 mo	EON in CKD
Kim et al., 2016 (26)	Korea	PC	Tertiary hospital	–	31	0	13–72§	52.0	Pulmonary	12 mo	Subclinical EON
Chen et al., 2015 (19)	Taiwan	RC	Medical center	2002– 2011	4,803	0	70.0 ± 14	74.0	Pulmonary	5.9 mo	EON outcomes
Li et al., 2014 (38)	China	RC	Tertiary hospital	1995– 2011	180	–	35.3 ± 14	8.0	NMO/TB M	28.5 mo	NMO- TB assoc
Chen et al., 2012 (20)	Taiwan	PB-CC	National database	1996– 2008	11,753	0	–	66.0	Pulmonary	–	EON risk factors
Davis et al., 2012 (39)	Multinational*	RCS	Multi-center	–	49	2	36‡	53.0	Ocular/S ystemic	12 mo‡	TB-ON
Hamade et al., 2010 (40)	Saudi Arabia	RC	Eye center	1997– 2007	49	4	45	57.0	Ocular	30.2 mo	Ocular TB Tx
Sinha et al., 2010 (41)	India	PC	Tertiary hospital	2008– 2009	101	0	30 ± 13	58.4	TBM	6 mo	TBM optic
Lee et al., 2008 (18)	Korea	RC	Tertiary hospital	2003– 2005	857	0	51.3 ± 17	57.0	Pulmonary	12.5 mo	EON recovery
Amitava et al., 2001 (42)	India	PC	Tertiary hospital	1995– 1996	100	100†	<24 mo: 48%	62.0	TBM	9 mo	Ped TBM ophth
Lamba et al., 1986 (43)	India	PC	Tertiary hospital	–	48	48†	1–3 y: 57%	58.0	TBM	4 mo	Ped TBM visual
Kalra et al., 1985 (44)	India	PC	Tertiary hospital	–	50	50†	Pediatric	–	TBM	6–24 mo	Ped TBM hydro

*Multinational study included 9 countries. †Exclusively pediatric cohort (<18 years). ‡Median value. §Range.

CKD, chronic kidney disease; CN, cranial nerve; CS, cross-sectional; EON, ethambutol optic neuropathy; Extrapulm, extrapulmonary; hydro, hydrocephalus; mo, months; N, number; NMO, neuromyelitis optica; -, not reported; ophth, ophthalmic; PB-CC, population-based case-control; PBC, population-based cohort; PC, prospective cohort; Ped, pediatric; PS, prospective surveillance Pulm, pulmonary; RC, retrospective cohort; RCS, retrospective case series; surv, surveillance; TB, tuberculosis; TB-ON, tuberculous optic neuropathy; TBM, tuberculous meningitis; Tx, treatment; y, years.

### 3.2 Risk of bias assessment

Risk of bias assessment using the NOS is summarized in **Supplementary Table 1**. Six studies (35%) were judged to have an overall low risk of bias, while 11 studies (65%) were assessed as having moderate risk of bias. No studies were judged to have a high risk of bias. The domains most frequently contributing to moderate risk ratings were comparability (mean score 0.8/2) and selection of participants (mean score 3.2/4). Studies with prospective designs and population-based sampling mainly demonstrated a lower risk of bias than retrospective hospital-based studies.

### 3.3 Pooled incidence of ethambutol optic neuropathy

**Figure 2:**
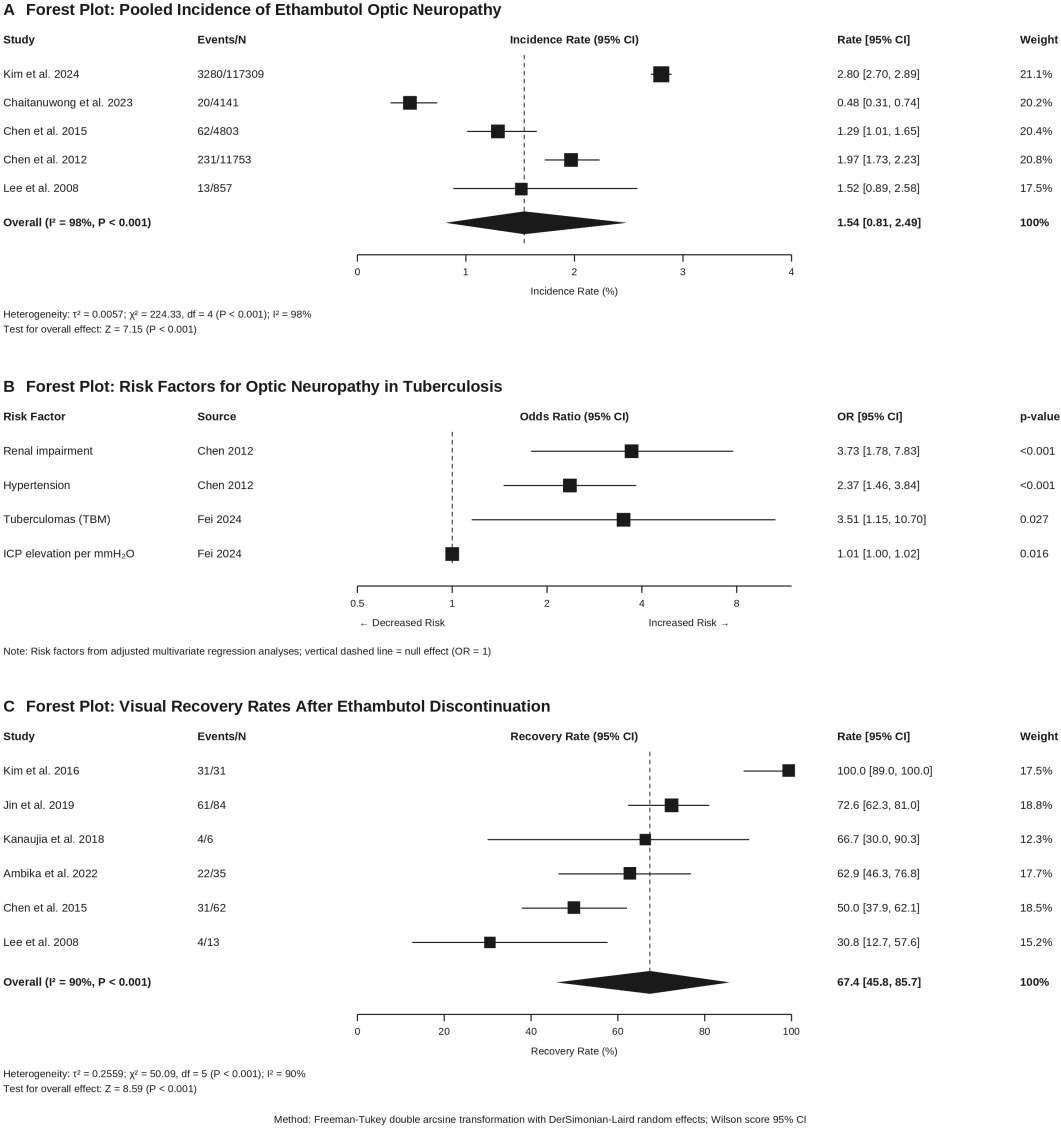
Forest plots showing **(A)** pooled EON incidence **(B)** risk factors for optic neuropathy in tuberculosis, and **(C)** visual recovery rates after ethambutol discontinuation.

Pooled EON incidence data from five studies, including 138,863 patients, are presented in **Table 2** and **Figure 2**. The analysis identified 3,606 EON events, resulting in a pooled incidence of 1.54% (95% CI: 0.81-2.49%, P-value <0.001. Significant heterogeneity was observed (I² = 98.2, τ² = 0.006), indicating between-study variation in reported incidence rates. Individual study incidence ranged from 0.48% to 2.80%. Subgroup analysis by study design revealed a higher incidence in the population-based studies (2.38%, 95% CI: 1.63-3.26%) compared to hospital-based studies (1.03%, 95% CI: 0.45-1.85%) (**Supplementary Table 2**).

**Table 2:** Pooled incidence of ethambutol optic neuropathy random-effects meta-analysis.

Study	Country	Design	Events	N	Incidence %	95% CI	Weight %	Mean dose (mg/kg/day)	Duration (mo)	I² (%)	τ²
Kim et al., 2024 (6)	Korea	PBC	3,280	117,309	2.80	2.70–2.89	21.1	–	6.4	—	—
Chaitanu wong et al., 2023 (21)	Thailand	RC	20	4,141	0.48	0.31–0.74	20.2	–	>6	—	—
Chen et al., 2015 (19)	Taiwan	RC	62	4,803	1.29	1.01–1.65	20.4	16.06 ± 4.3	5.94	—	—
Chen et al., 2012 (20)	Taiwan	PB-CC	231	11,753	1.97	1.73–2.23	20.8	–	>3	—	—
Lee et al., 2008 (18)	Korea	RC	13	857	1.52	0.89–2.58	17.5	17.85 ± 2.2	9.38	—	—
Pooled (RE)	—	—	3,606	138,863	1.54	0.81–2.49	100.0	—	—	98.2	0.006

CI, confidence interval; I², percentage of variability due to heterogeneity; mo, months; N, number; -, not reported; PB-CC, population-based case-control; PBC, population-based cohort; RC, retrospective cohort; RE, random effects; τ², between-study variance; Q = 224.3, df = 4, P-value< 0.001; 95% prediction interval: 0.18–4.18%.

**Figure 3:**
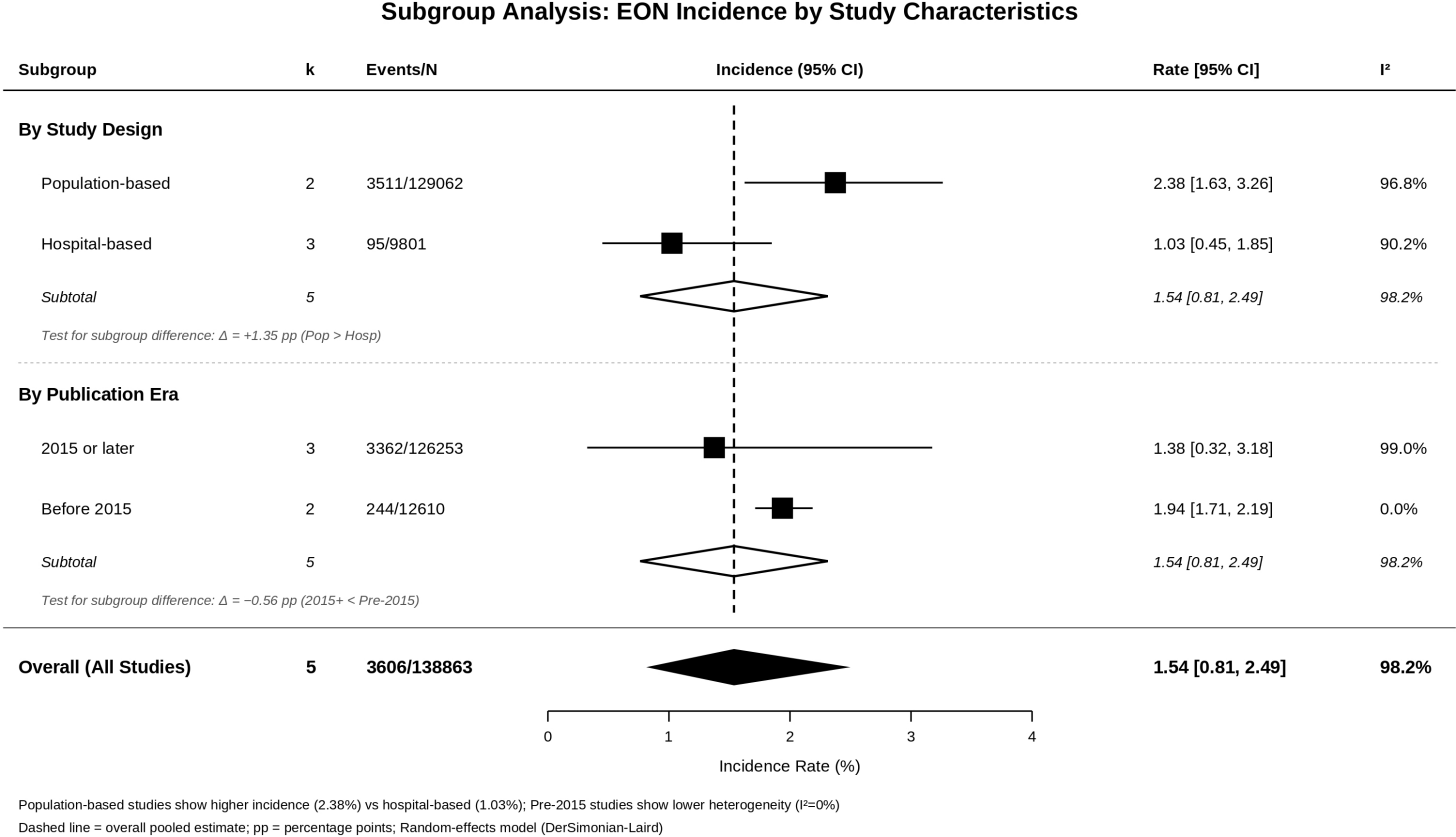
Subgroup analysis of EON incidence by study design and publication era.

Subgroup analysis by publication era showed similar estimates for studies conducted before 2015 (1.94%) and those conducted within or after 2015 (1.38%), with no significant subgroup difference (**Figure 3**). Subgroup analysis by dose showed as significantly higher incidence in patients receiving ≥ 15 mg/kg/day (68.0%; 95% CI: 0.49-0.83) compared to those receiving <15mg/kg/day (17.0%; 95% CI: 0.09-0.26), indicating a clear dose-dependent risk. Subgroup analysis by treatment duration showed higher incidences in patients treated for more than 6 months (2.1%; 95% CI: 0.00-1.00) compared to patients treated for less than 6 months (0.9%; 95% CI: 0.01-0.66) (**Supplementary Figure 3**). Leave-one-out sensitivity analysis demonstrated that no single study disproportionately influenced the pooled estimate (**Supplementary Figure 1**). Excluding Kim et al., 2024 (33), which contributed the largest sample, resulted in a pooled incidence of 1.26% (95% CI: 0.61-2.13%).

A meta-regression analysis was performed to assess the impact of variables such as treatment duration, screening practices, geographical region and study design on heterogeneity. The meta-regression results are presented in **Supplementary Figure 4** shows that none of the tested moderators namely study design, duration and publication era reached conventional statistical significance. However, prospective design approached borderline significance (p = 0.0576. Residual heterogeneity remained extremely high (I2 = 97.9%) indicating that much of the variability is unexplained by these moderators. This highlights that the pooled estimates should be interpreted with caution and indicate that other factors such as ethambutol dose or screening practices could be contributing to the heterogeneity.

### 3.4 Risk factors for ethambutol optic neuropathy

**Figure 4:**
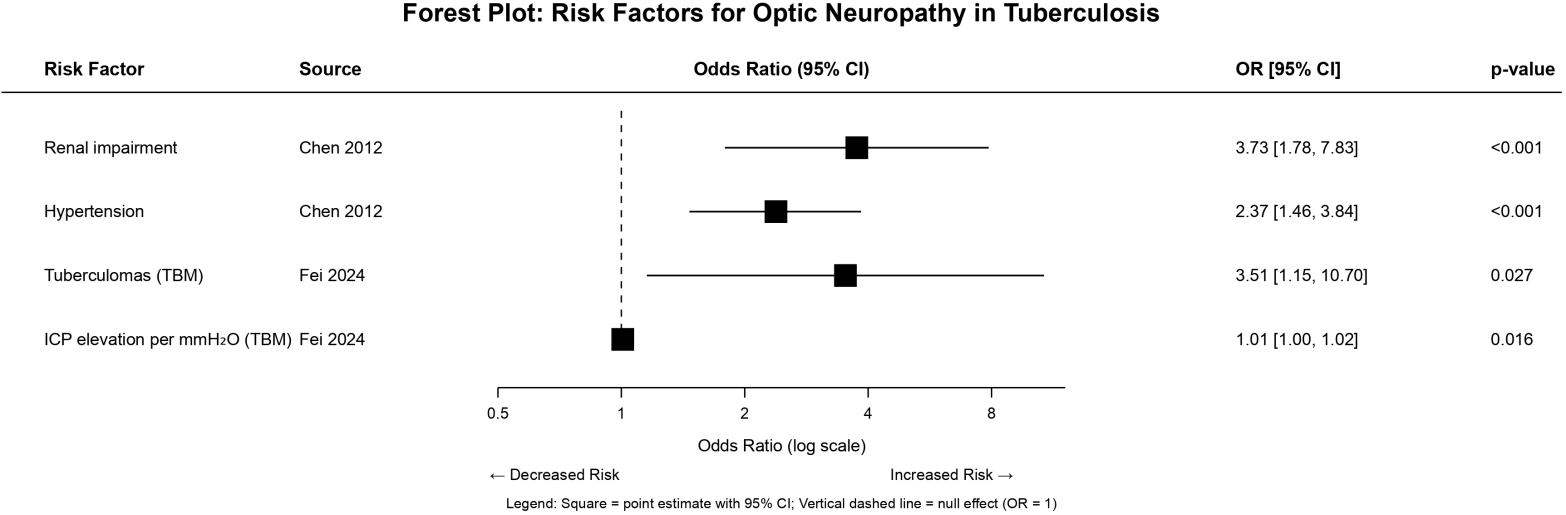
Forest plot of odds ratios for risk factors associated with optic neuropathy in tuberculosis and tuberculous meningitis.

Risk factor analysis from available studies is presented in **Table 3** and **Figure 4**. Renal impairment was the strongest identified risk factor for EON development (OR 3.73, 95% CI: 1.78-7.83, P-value<0.001), with a number needed to harm (NNH) of 26 patients. Hypertension also demonstrated significant association (OR 2.37, 95% CI: 1.46-3.84, P-value<0.001, NNH = 48). Ethambutol dose categories (800–1199 mg/day and >1200 mg/day) showed non-significant trends toward increased risk (OR 1.21 and 1.41, respectively, P-values>0.05). For TBM-associated optic neuropathy, intracranial tuberculomas (OR 3.51, 95% CI: 1.15-10.70, P-value= 0.027) and elevated intracranial pressure (OR 1.011 per mmH_2_O, P-value= 0.012) were significant predictors.

**Table 3:** Risk factors for optic neuropathy in tuberculosis quantitative effect size analysis.

Risk factor	Condition	Study	N	Effect measure	Effect size	95% CI	SE (log)	Z	P-value	NNH
Renal impairment	EON	Chen et al., 2012 (20)	11,753	OR	3.73	1.78–7.83	0.378	3.48	<0.001	26
Hypertension		Chen et al., 2012 (20)	11,753	OR	2.37	1.46–3.84	0.247	3.50	<0.001	48
Dose >1200 mg/day		Chen et al., 2012 (20)	11,753	OR	1.41	0.89–2.24	0.235	1.46	0.14	—
Dose 800– 1199 mg/day		Chen et al., 2012 (20)	11,753	OR	1.21	0.83–1.78	0.195	0.98	0.32	—
Tuberculomas	TBM-ONP	Fei et al., 2024 (32)	250	OR	3.51	1.15–10.70	0.570	2.21	0.027	22
ICP (per mmH_2_O)		Fei et al., 2024 (32)	250	OR	1.011	1.002–1.020	0.004	2.51	0.016	—
CSF protein (g/L)		Fei et al., 2024 (32)	250	MD	0.35	0.02–0.68	—	2.07	0.039	—
CSF protein >75 mg%	TBM-OA	Lamba et al., 1986 [PED] (43)	48	Assoc	—	—	—	—	0.001	—

Assoc, association; CI, confidence interval; CSF, cerebrospinal fluid; EON, ethambutol optic neuropathy; ICP, intracranial pressure; MD, mean difference; N, number; NNH, number needed to harm; OA, optic atrophy; ONP, optic nerve palsy; OR, odds ratio; PED, pediatric; SE, standard error; TBM, tuberculous meningitis; Z, z-score.

### 3.5 Neuro-ophthalmic manifestations of tuberculous meningitis

TBM neuro-ophthalmic manifestations from six studies enrolling 1,035 patients are presented in **Table 4**. Pooled prevalence estimates using GLMM demonstrated cranial nerve III palsy in 17.4% (95% CI: 4.2-37.0%), cranial nerve VI palsy in 2.6% (95% CI: 0.1-8.2%), papilledema in 12.5% (95% CI: 2.6-28.1%), and optic atrophy in 16.7% (95% CI: 4.2-35.0%). Hydrocephalus was present in 41.5% (95% CI: 5.0-85.2%) of TBM patients, and overall mortality was 15.6% (95% CI: 4.8-31.1%). Significant heterogeneity was observed across all manifestations (I² ranging from 91.4% to 99.2%).

**Table 4:** Neuro-ophthalmic manifestations in tuberculous meningitis GLMM proportional meta-analysis.

Study	Country	Population		N	CN III Palsy n/N (%)	CN VI Palsy n/N (%)	Papilledema n/N (%)		Optic Atrophy n/N (%)	Hydrocephalus n/N (%)	Mortality n/N (%)
Fei et al., 2024 (32)	China	Adult		250	1/250 (0.4)	1/250 (0.4)	–		–	–	34/250 (13.6)
Li et al., 2019 (23)	China			486	41/486 (8.4)	3/486 (0.6)	–		–	11/486 (2.3)	7/486 (1.4)
Sinha et al., 2010 (41)	India			101	52/101 (51.5)*	–	31/101 (30.7)		6/101 (5.9)	31/101 (30.7)	13/101 (12.9)
Amitava et al. 2001 (42)	India	Pediatric		100	18/100 (18.0)	11/100 (11.0)	3/100 (3.0)		7/100 (7.0)	70/100 (70.0)	30/100 (30.0)
Lamba et al. 1986 (43)	India			48	13/48 (27.1)	–	3/48 (6.3)		7/48 (14.6)	–	15/48 (31.3)
Kalra et al. 1985 (44)	India			50	–	–	7/50 (14.0)		24/50 (48.0)	39/50 (78.0)	–
**Pooled**	—	All (k=4–5)		1,035	**17.4 [4.2–37.0]**	**2.6 [0.1– 8.2]**	**12.5 [2.6–28.1]**		**16.7 [4.2–35.0]**	**41.5 [5.0–85.2]**	**15.6 [4.8–31.1]**
**Overall** **[95% CI]**											
**Pooled Adult [95% CI]**	—	Adult		837	**14.3 [0.4–42.5]**	**0.7 [0.2–1.4]**	**30.7 [22.5–40.3]**		**5.9 [2.8–12.4]**	**13.0 [0.3–51.0]**	**8.2 [1.0–21.3]**
**Pooled** **Pediatric** **[95% CI]**	—	Pediatric		198	**21.8 [13.7–31.0]**	**11.0 [6.3–18.6]**	**7.4 [2.4–15.0]**		**21.2 [3.5–48.3]**	**72.5 [65.0–79.4]**	**30.7 [23.5–38.3]**
**I² (%)**	—	—		—	97.7	91.8	91.4		92.6	99.2	96.6
**RR**	—	—		—	1.52 [1.05–2.19]	15.7 [4.8–51.4]	0.24 [0.13–0.44]		3.57 [1.56–8.16]	5.56 [4.09–7.56]	3.75 [2.63–5.35]
**Ped/Adult** **[95% CI]**											

CI, confidence interval; CN, cranial nerve; GLMM, generalized linear mixed model; I², heterogeneity statistic; k, number of studies; N, number; -, not reported; Ped, pediatric; RR, risk ratio. *Total cranial nerve involvement reported.

For Adult versus pediatric subgroup analysis revealed significant differences in manifestation patterns. Pediatric patients demonstrated significantly higher rates of hydrocephalus (72.5% vs 13.0%, RR 5.56, 95% CI: 4.09-7.56), cranial nerve VI palsy (11.0% vs 0.7%, RR 15.7, 95% CI: 4.8-51.4), optic atrophy (21.2% vs 5.9%, RR 3.57, 95% CI: 1.56-8.16), and mortality (30.7% vs 8.2%, RR 3.75, 95% CI: 2.63-5.35). In contrast, adult patients showed higher papilledema rates (30.7% vs 7.4%, RR 0.24, 95% CI: 0.13-0.44). Medical Research Council grade distribution and additional clinical parameters are reported in **Supplementary Table 5**.

### 3.6 Subclinical neurotoxicity detection

**Figure 5:**
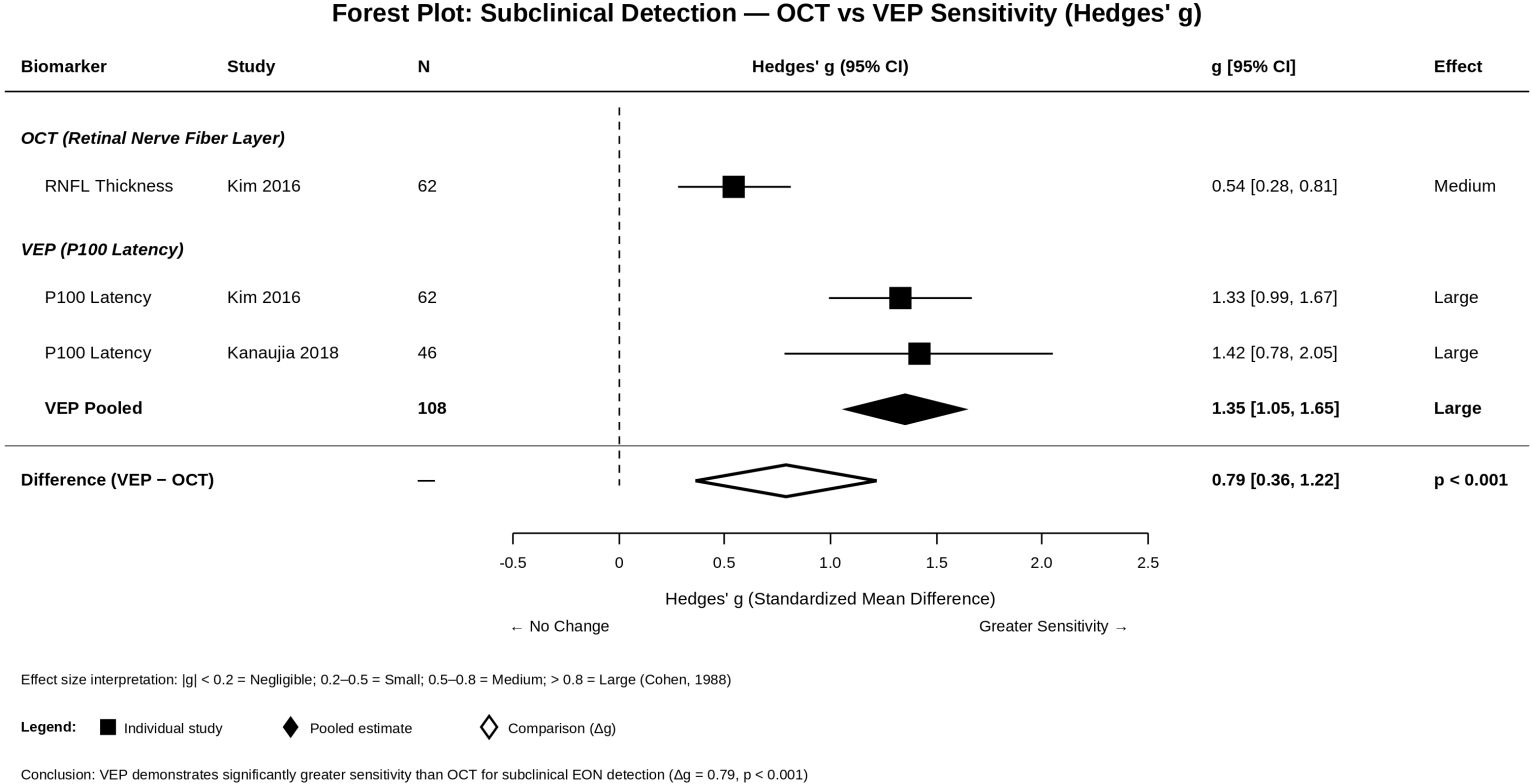
Forest plot comparing subclinical detection sensitivity of VEP versus OCT.

Subclinical neurotoxicity biomarker analysis from three studies is presented in **Table 5** and **Figure 5**. VEP P100 latency prolongation demonstrated large effect sizes for subclinical detection (Hedges’ g=1.106, 95% CI: 0.790-1.422, P-value<0.0001 in Kim et al., 2016 (26); Hedges’ g= 1.391, 95% CI: 0.818-1.965, P-value<0.0001 in Kanaujia et al., 2018) (37). OCT-measured retinal nerve fiber layer (RNFL) thickness changes showed small effect sizes (Hedges’ g= 0.420, 95% CI: 0.161-0.680, P-value=0.0015). Direct comparison revealed that VEP demonstrated higher detection of subclinical changes rather than OCT (Hedges’ g difference = 0.686, 95% CI: 0.276-1.096, P-value = 0.001). Combined VEP and OCT monitoring achieved 67% detection rate for subclinical changes.

**Table 5:** Subclinical neurotoxicity detection standardized mean difference meta-analysis.

Study	Biomarker	N (eyes)	Baseline (Mean ± SD)	Follow-up (Mean ± SD)	Mean Δ	Hedges’ g	95% CI	Z	P-value	Effect Size
Kim et al., 2016 (26)	RNFL thickness (OCT)	62	103.5 ± 9.3 μm	107.5 ± 9.5 μm	+4.0 μm	0.420	0.161–0.680	3.17	0.0015	Small
Jin et al. 2019 (28)		168	106.1 ± 14.1 μm	—	+3.7 μm	—	—	—	–	Small (est)
Kim et al., 2016 (26)	P100 latency (VEP)	62	104.5 ± 5.6 ms	112.3 ± 8.1 ms	+7.8 ms	1.106	0.790–1.422	6.86	<0.0001	Large
Kanaujia et al. 2018 (37)		46	108.6 ± 6.8 ms	100.0 ± 5.0 ms†	+8.6 ms	1.391	0.818–1.965	4.76	<0.0001	Large
Lee et al., 2008 (18)	VEP detection rate	13	—	—	—	—	38.1–85.8%	—	—	65.4%
	VF detection rate	13	—	—	—	—	38.1–85.8%	—	—	65.4%
	Color vision detection	13	—	—	—	—	35.0–82.8%	—	—	61.5%
Kanaujia et al. 2018 (37)	Pre-clinical VEP rate	6	—	—	—	—	18.8–81.2%	—	—	50.0%
Kim et al., 2016 (26)	Combine d OCT+VE P rate	31	—	—	—	—	48.8–81.4%	—	—	67.0%
**VEP vs OCT** **compari son**	**Δg (VEP−** **OCT)**	**62**	**—**	**—**	**—**	**0.686**	**0.276–1.096**	**3.29**	**0.001**	**VEP > OCT**

CI, confidence interval; Δ, change; est, estimated; g, Hedges’ g standardized mean difference; ms, milliseconds; N, number; -, not reported; OCT, optical coherence tomography; P100, positive peak at 100 ms; RNFL, retinal nerve fiber layer; SD, standard deviation; VEP, visual evoked potential; VF, visual field; Z, z-score; μm, micrometers. †Normal reference value for comparison.

### 3.7 Visual outcomes and recovery

Visual outcome data are presented in **Table 6**. Pooled visual recovery rate in clinical EON across four studies (N = 116) was 52.4% (95% CI: 40.4-64.1%, I²=30.9%). Recovery rates varied by disease severity, with subclinical EON showing 100% recovery, while severe clinical EON demonstrated, only 30.7% recovery. Factors associated with improved recovery included younger age (MD= -3.8 years, P-value= 0.095) and earlier ethambutol discontinuation. For TBM-related visual impairment, adjunctive corticosteroid therapy demonstrated a significant protective effect (8% vs 32% visual impairment, NNT = 4.2, P-value= 0.04).

**Table 6:** Visual outcomes and therapeutic interventions in tuberculosis-related optic neuropathy.

Study	Condition	Outcome measure	N	Events/Rate	95% CI (%)	Follow-up	Effect size	P-value	Notes
Kim et al., 2016 (26)	Subclinical EON	Visual Recovery	31	31/31 (100.0%)	89.0–100.0	6 months	—	—	All subclinical, no VA loss
Jin et al., 2019 (28)			84	61/84 (73.0%)	62.3–81.0	12 months	—	—	RNFL normalized in most
Kanaujia et al., 2018 (37)	Clinical EON (CKD)	Visual Recovery	6	4/6 (66.7%)	30.0–90.3	6 months	—	—	CKD population
Ambika et al., 2022 (34)	Clinical EON	Visual Recovery	35	22/35 (62.9%)	46.3–76.8	Variable	—	—	Tertiary center
Chen et al., 2015 (19)			62	31/62 (50.0%)	37.9–62.1	Variable	—	—	Population-based
Lee et al., 2008 (18)			13	4/13 (30.7%)	12.7–57.6	12 months	—	—	Severe cases
Pooled Clinical EON (RE)	Clinical EON	Visual Recovery	116	61/116 (52.4%)	40.4–64.1	—	I²=30.9%	—	Random-effects pooled
Hamade et al., 2010 (40)	TBM	Visual Impairment (Steroid)	~100	8% vs 32%	—	12 months	NNT=4.2	0.04	Steroid protective
Kim et al., 2024 (33)		1-Year Cumulative Incidence	117,309	2.1%	—	12 months	—	—	Population -based
		2-Year Cumulative Incidence	117,309	2.8%	—	24 months	—	—	Population-based
		1-Year Visual Impairment	117,309	0.4%	—	12 months	—	—	Severe outcome
		1-Year Blindness	117,309	0.08%	—	12 months	—	—	Rare but devastating
Chen et al., 2015 (19)		Age (Improved vs Non-improved)	62	74.8 vs 78.6 years	—	—	MD=−3.8 years	0.095	Trend: younger = better recovery
Lee et al., 2008 (18)		Time to EMB Discontinu ation	13	5.8 months (mean onset)	—	—	—	—	Earlier stop = better outcome

CI, confidence interval; CKD, chronic kidney disease; EMB, ethambutol; EON, ethambutol optic neuropathy; I², heterogeneity statistic; MD, mean difference; N, number; NNT, number needed to treat; RE, random effects; RNFL, retinal nerve fiber layer; TBM, tuberculous meningitis; VA, visual acuity.

Differential diagnostic features distinguishing EON from tuberculous optic neuropathy (TB-ON) are summarized in **Supplementary Table 3**. EON was characterized by bilateral involvement (94.3% vs 37.8%), disc pallor (80.0% vs 21.6%), and red-green dyschromatopsia (85.7% vs 32.4%), while TB-ON was associated with pain on eye movement (40.5% vs 0%), disc edema (59.5% vs 11.4%), and steroid responsiveness (75.7% vs minimal).

### 3.8 Publication bias and certainty of evidence

Publication bias assessment is presented in **Supplementary Figure 2** and **Supplementary Table 4**. Funnel plot visual inspection for EON incidence showed some asymmetry, with Egger’s regression demonstrating significant asymmetry (intercept = -8.476, P-value<0.001), while Begg’s rank correlation showed no significant asymmetry (τ= -0.600, P-value= 0.233). The limited number of studies (k=5) precluded trim-and-fill adjustment.

GRADE assessment rated the certainty of evidence as moderate for EON incidence (downgraded for inconsistency), moderate for EON risk factors (downgraded for indirectness), very low for TBM manifestations (downgraded for risk of bias, inconsistency, and imprecision), moderate for subclinical biomarkers (downgraded for indirectness), and low for visual recovery (downgraded for risk of bias and imprecision).

## 4 Discussion

This systematic review and meta-analysis provide a detailed evaluation of neuro-ophthalmic complications associated with tuberculosis and its treatment, encompassing EON incidence and risk factors, TBM manifestations across age groups, subclinical biomarker utility, and visual recovery outcomes. Our findings have demonstrated that EON occurs in approximately 1.54% of patients receiving ethambutol-containing regimens, with renal impairment and hypertension representing significant modifiable risk factors. In addition to that, we identified important age-related differences in TBM neuro-ophthalmic manifestations and demonstrated the higher sensitivity of VEP over OCT for subclinical toxicity detection.

While EON, TBM-related neuro-ophthalmic complications and subclinical biomarkers represent different clinical categories with distinct pathophysiological mechanisms, they are presented within a single meta-analytic framework in this review. Each category is analyzed separately, including pooled estimates and subgroup analysis to identify their clinical differences. Combining the outcomes offers a comprehensive overview of the TB-associated neuro-ophthalmic outcomes by understanding the full spectrum of neuro-ophthalmic risk, manifestations and monitoring. The high heterogeneity limited direct comparisons between the categories.

Our pooled EON incidence of 1.54% (95% CI: 0.81-2.49%) aligns with previously reported estimates in the literature. Two of the largest epidemiologic studies investigating EON to date showed the prevalence of EON in all patients taking ethambutol to be between 0.7 and 1.29%, a value consistent with previous reports (16). The pooled cumulative incidence of any visual impairment in all patients was 22.5 per 1000 persons treated with EMB (95% CI 10.2-35), and permanent impairment was 4.3/1000 (95% CI 0.3-9.0) (17). The incidence of EON in Koreans is estimated to be less than 2% (18). A 10-year experience from southern Taiwan found an incidence of 1.29% (19). The significant heterogeneity observed in our analysis (I²=98.2%) likely reflects differences in diagnostic criteria, screening practices, and population characteristics across studies. Our subgroup analysis revealed higher incidence in population-based studies compared to hospital-based studies, suggesting possible under ascertainment in settings without systematic screening protocols. Variability in diagnostic criteria for EON and TBM-related complications likely contributed to significant heterogeneity (I2 >90%). Some studies incorporated clinical examination, while others incorporated standardized measures such as VE or imaging assessment. The inconsistency in diagnostic criteria as shown in **Table 7** limits the comparability of incidence estimates and highlights the need for standardized outcome definitions in future studies.

**Table 7:** Diagnostic criteria used in included studies.

Study	Diagnostic approach/Criteria	Screening modalities Used	Subclinical detection
Kim et al., 2024 (33)	Incidence-based (database diagnosis codes)	VA, VF, OCT, Color	Not specified
Kim et al., 2016 (26)	Prospective monitoring protocol	OCT, VEP, Color, VF	Yes
Chen et al., 2015 (19)	Clinical diagnosis	VA	No
Chen et al., 2012 (20)	Administrative coding (case-control)	VA	No
Lee et al., 2008 (18)	Clinical + electrophysiology	VA, VEP, Color, VF	Limited
Chaitanuwong et al., 2023 (21)	Clinical diagnosis	VA, Color, VF	Not specified
Ambika et al., 2022 (34)	Clinical features + imaging	VA, VF, OCT, MRI	Not specified
Shirley et al., 2020 (36)	Surveillance-based reporting	VA, Color, VF	Not specified
Jin et al., 2019 (28)	Subclinical detection focus	OCT, VF	Yes
Kanaujia et al., 2018 (37)	Clinical + electrophysiology	VEP, Color	Partial

The identification of renal impairment (OR 3.73) and hypertension (OR 2.37) associated with increased likelihood of EON development has important implications for management. These findings agree with previous studies identifying similar associations. A nationwide population-based study from Taiwan found that EON patients were at risk of hypertension (adjusted OR = 1.62, 95% CI 1.16 to 2.26) and renal diseases (with ESRD, adjusted OR = 3.73, 95% CI 1.79 to 7.74) (20). Hypertension was associated with EON, and the effect was hypothesized to be mediated through reduced renal function (21). The association with renal impairment is biologically plausible, given that ethambutol is primarily excreted through the kidneys, and reduced clearance would lead to elevated drug levels concentrations and increased toxicity risk. Our calculated NNH of 26 for renal impairment suggests that targeted screening in this high-risk population could prevent a significant proportion of EON cases. Higher daily and cumulative doses of ethambutol were associated with a higher risk of optic neuropathy (**Table 8**). Studies reported doses above 15 mg/kg per day or cumulative dose exceeding 2800 mg/kg/day, suggesting higher incidence rate. Longer treatment duration was associated with high risk, indicating the dose-dependent nature of ethambutol neurotoxicity.

**Table 8:** Dose-response table.

Study	N (EMB)	Daily dose (mg/kg)	Dose range	Cumulative dose (mg/kg)	Duration (months)	EON events	EON incidence (%)	OR/RR (if reported)
Kim 2024 (33)	117,309	–	–	–	6.4	3,280	2.8	–
Jin 2019 (28)	84	14.72 ± 3.07	–	–	6.7	14	16.7	–
Kanaujia 2018 (37)	23	15–25	15–25	–	3	6	26	3.73
Chen 2015 (19)	4,803	16.06 ± 4.3	–	2,820 ± 2,157	5.94	62	1.29	–
Lee 2008 (18)	857	17.85 ± 2.21	–	–	9.38	13	1.50	–

Regarding TBM neuro-ophthalmic manifestations, our pooled estimates demonstrated cranial nerve III palsy in 17.4%, cranial nerve VI palsy in 2.6%, papilledema in 12.5%, and optic atrophy in 16.7% of the TBM patients. In contrast with other studies, Sharma et al. (22) stated that 60 (38%) patients had cranial neuropathy, with the abducent nerve being the most frequently (32.3%) affected cranial nerve. Out of 486 patients evaluated, 72 patients (14.8%) were diagnosed as TBM with cranial nerve palsy (23). Cranial nerve palsy was observed in approximately 33.3% of TBM patients (24). As reported in the literature, cranial nerve palsy is considered a massive complication that could arise from TBM. However, the variability in the reported frequency likely reflects differences in severity, stage at presentation and underlying pathophysiological mechanism. Our analysis revealed important age-related differences, with pediatric patients demonstrating significantly higher hydrocephalus rates (72.5% vs 13.0%, RR 5.56), optic atrophy (21.2% vs 5.9%, RR 3.57), and mortality (30.7% vs 8.2%, RR 3.75) compared to adults. These differential vulnerability patterns may reflect the greater susceptibility of the developing nervous system to inflammatory injury, the higher proportion of severe disease at presentation in pediatric populations, or differences in healthcare access and diagnostic delays.

Our finding that VEP demonstrates effective detection for subclinical EON over OCT (Hedges’ g Difference = 0.686, P-value = 0.001) has significant implications for screening strategies. In five of the six cases with VEP changes, alterations were not associated with a change in visual function as measured by clinical neuro-ophthalmologic examination, confirming the usefulness of VEPs in the detection of subclinical optic nerve disease (25). Pattern VEP and retinal nerve fiber layer (RNFL), OCT is a suitable test for the early detection of subclinical ethambutol-induced ocular toxicity (26). P100 latency was delayed in 34.8% of cases, and recording of VEP is a significantly useful objective test for subclinical optic nerve damage (27). Visual field and OCT are more sensitive for the detection of subtle changes compared to clinical examination (28). Subclinical damage in the form of an increase in VER latency was seen in 46% of eyes, while the incidence of clinical EON optic neuropathy was less than 2% (29). The large effect sizes observed for VEP P100 latency prolongation (Hedges’ g= 1.106-1.391) suggest that electrophysiological testing may detect functional impairment before structural changes become apparent on OCT. However, implementation challenges, including cost, availability, and technical expertise may limit widespread adoption of VEP monitoring, particularly in resource-limited settings where the tuberculosis burden is highest.

The pooled visual recovery rate of 52.4% (95% CI: 40.4-64.1%) in clinical EON focuses on both the reversibility of early disease and the importance of timely diagnosis. Visual function after discontinuation of ethambutol is reversible in only a minority of patients and does not usually occur if optic disc pallor and atrophy are present (18). Half of the patients showed visual improvement after discontinuation of ethambutol (19). After stopping ethambutol, the visual function of 60% of patients improved by at least two lines on the Snellen chart, with a mean recovery time of 6.9 months (21). Our observation that subclinical EON demonstrated almost complete recovery, while severe clinical EON showed only 30.7% recovery, highlighting the importance of early detection through systematic screening programs.

Several limitations warrant consideration. First, the significant heterogeneity observed across studies for most outcomes limits the precision of pooled estimates. Second, the absence of RCTs and predominance of retrospective designs introduces possible selection and ascertainment biases. Third, the limited number of studies reporting subclinical biomarker data precluded detailed subgroup analysis analyses for this outcome. Fourth, the publication bias assessment was limited by the small number of studies available for certain outcomes. Fifth, possible inaccuracy from the conversion of medians with interquartile ranges to means and standard deviations. Sixth, the geographic concentration of studies in Asian populations may limit generalizability to other regions. Finally, this review was restricted to studies published in English, which may have introduced language bias, potentially excluding relevant evidence published in other languages affected the generalizability of the findings.

Despite these limitations, our findings have important implications for practice. First, patients with renal impairment and hypertension should be considered high-risk for EON and may benefit from enhanced monitoring or alternative regimens when feasible. Second, VEP should be considered for subclinical detection in settings where available, particularly for high-risk patients. Third, pediatric TBM patients require intensive neuro-ophthalmic monitoring, given their differential vulnerability to visual sequelae. Fourth, early recognition and prompt ethambutol discontinuation remain the cornerstone of EON management, as recovery rates decline significantly with disease progression.

## 5 Conclusion

In conclusion, this systematic review and meta-analysis demonstrated that EON occurs in approximately 1.54% of patients receiving ethambutol, with renal impairment and hypertension represents significant modifiable risk factors warranting enhanced surveillance. TBM-related neuro-ophthalmic complications demonstrated important age-related differences, with pediatric patients experienced significantly higher rates of hydrocephalus, optic atrophy, and mortality compared to adults. Although VEP appeared more sensitive than OCT in detecting subclinical changes, Clinical applicability is limited by the small number of studies and methodological heterogeneity. Visual recovery occurred in approximately half of clinical EON cases, with outcomes strongly dependent on early recognition and prompt ethambutol discontinuation. These findings support implementation of risk-stratified screening protocols targeting high-risk populations, particularly patients with renal dysfunction and hypertension. Future studies need to evaluate the cost-effectiveness of VEP-based screening strategies in TB-endemic settings and investigate novel therapeutic approaches for established optic neuropathy.

## Data Availability

The raw data supporting the conclusions of this article will be made available by the authors, without undue reservation.
