# Supplementary figures and images for: Neuro-ophthalmic complications of tuberculosis and its treatment: a systematic review and meta-analysis
Source: Front Ophthalmol (Lausanne). 2026 May 29;6:1818640. doi: 10.3389/fopht.2026.1818640 (PMC13259741; doi:10.3389/fopht.2026.1818640)

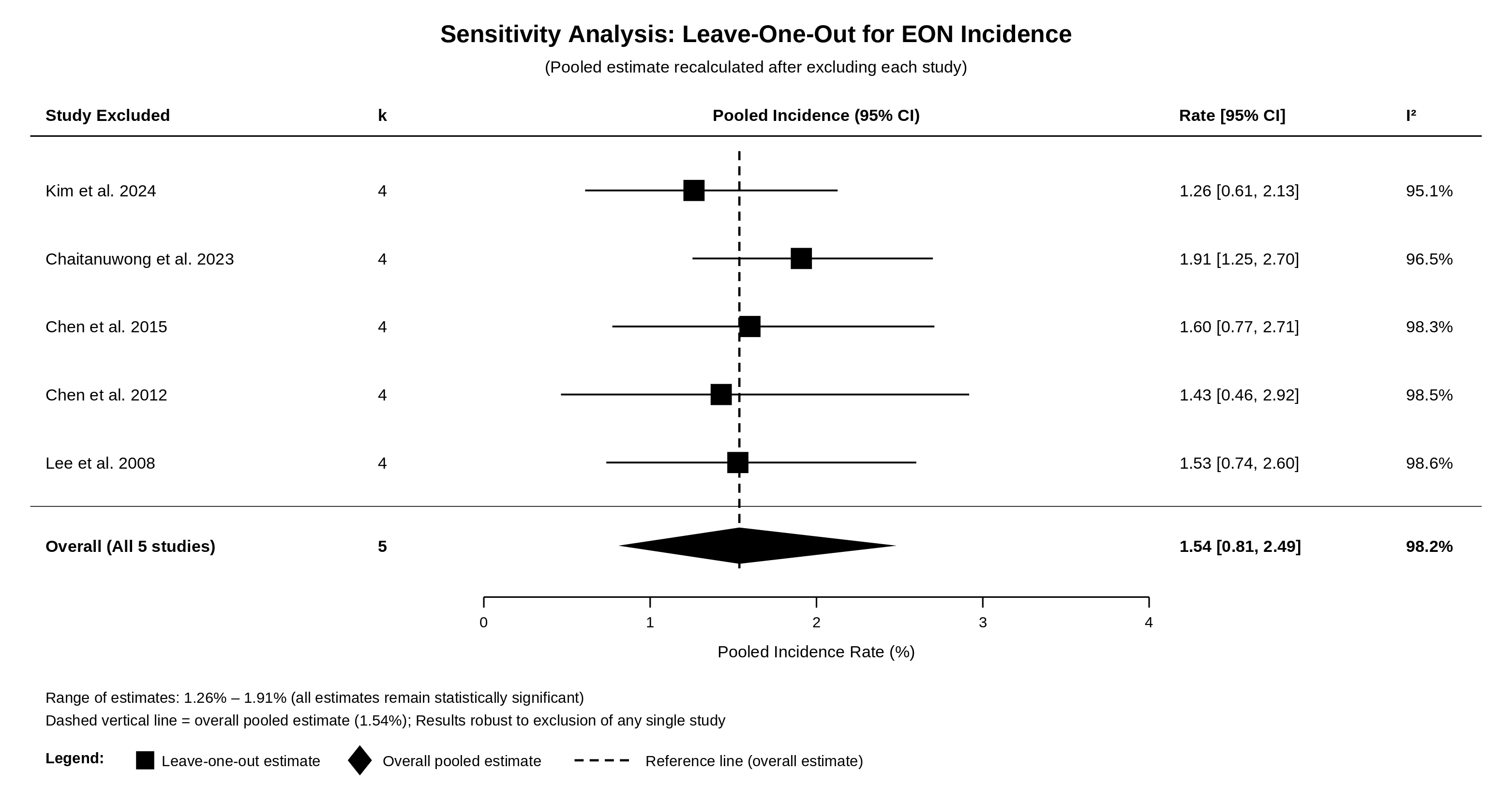

Supplement: Supplementary file 1 [file Image1.jpeg]

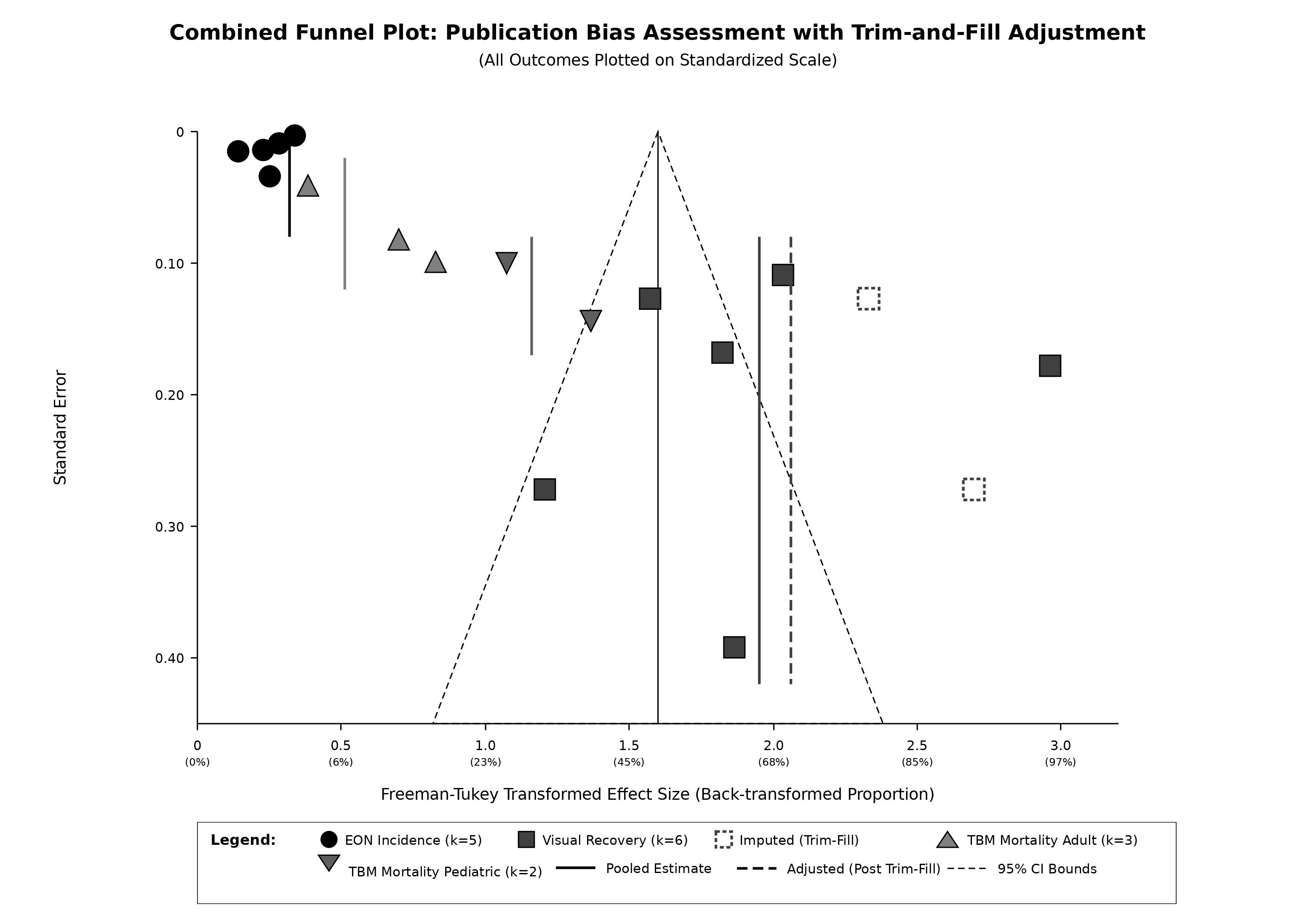

Supplement: Supplementary file 2 [file Image2.jpeg]

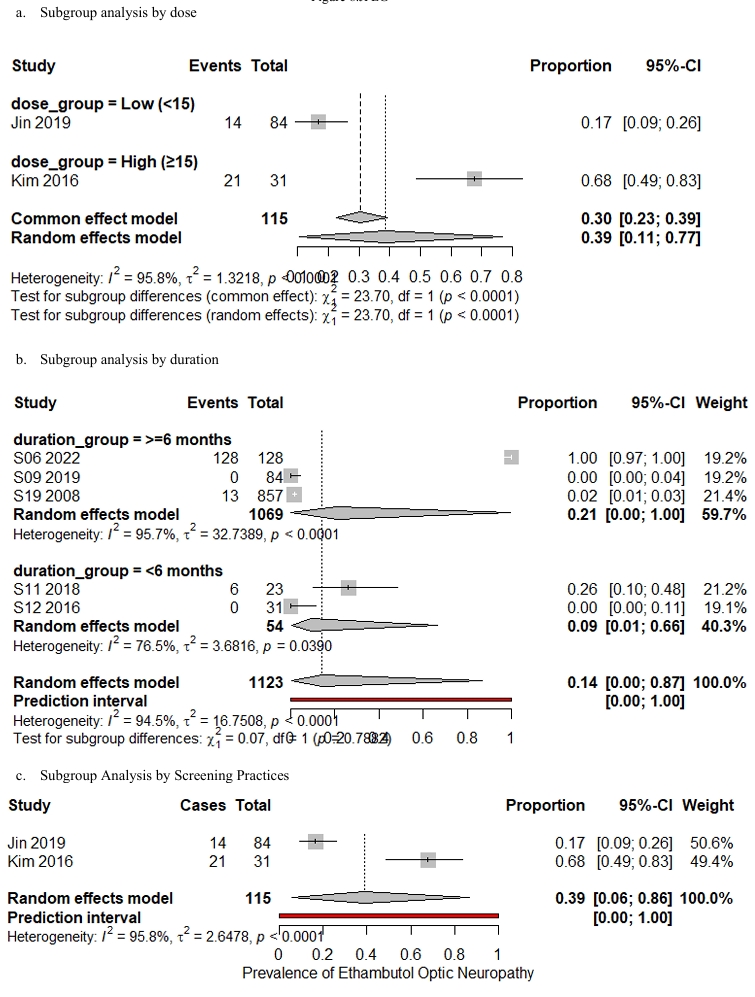

Supplement: Supplementary file 3 [file Image3.jpeg]

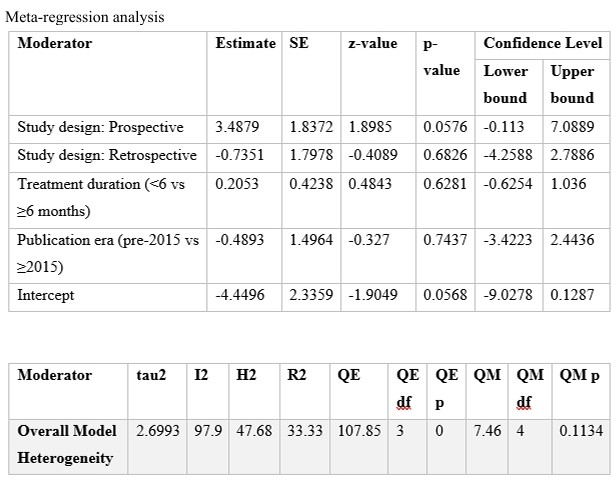

Supplement: Supplementary file 4 [file Image4.jpeg]
