# Supplementary material for: Neuro-ophthalmic complications of tuberculosis and its treatment: a systematic review and meta-analysis
Source: Front Ophthalmol (Lausanne). 2026 May 29;6:1818640. doi: 10.3389/fopht.2026.1818640 (PMC13259741; doi:10.3389/fopht.2026.1818640)
Supplement: Supplementary file 5 [file Table1.docx]

**Supplementary Table 1:** Risk of Bias Assessment Using Newcastle-Ottawa Scale.

| **Study** | **Design** | **Focus** | **Selection (S1-S4)** | **Comparability (C1-C2)** | **Outcome (O1-O3)** | **Total Score** | **Risk of Bias** |
| --- | --- | --- | --- | --- | --- | --- | --- |
| Kim et al. 2024 | PBC | EON | ★★★★ (4/4) | ★★ (2/2) | ★★★ (3/3) | 9/9 | Low |
| Chaitanuwong et al. 2023 | RC | EON | ★★★☆ (3/4) | ☆☆ (0/2) | ★★☆ (2/3) | 5/9 | Moderate |
| Chen et al. 2015 | RC | EON | ★★★☆ (3/4) | ★☆ (1/2) | ★★☆ (2/3) | 6/9 | Moderate |
| Chen et al. 2012 | PB-CC | EON Risk | ★★★★ (4/4) | ★★ (2/2) | ★★★ (3/3) | 9/9 | Low |
| Lee et al. 2008 | RC | EON | ★★★☆ (3/4) | ☆☆ (0/2) | ★★☆ (2/3) | 5/9 | Moderate |
| Kim et al. 2016 | PC | Subclinical EON | ★★★☆ (3/4) | ★☆ (1/2) | ★★★ (3/3) | 7/9 | Low |
| Jin et al. 2019 | PC | Subclinical EON | ★★★☆ (3/4) | ★☆ (1/2) | ★★★ (3/3) | 7/9 | Low |
| Kanaujia et al. 2018 | PC | EON in CKD | ★★★☆ (3/4) | ☆☆ (0/2) | ★★☆ (2/3) | 5/9 | Moderate |
| Fei et al. 2024 | RC | TBM-ONP | ★★★☆ (3/4) | ★★ (2/2) | ★★☆ (2/3) | 7/9 | Low |
| Li et al. 2019 | RC | TBM | ★★★☆ (3/4) | ★☆ (1/2) | ★★☆ (2/3) | 6/9 | Moderate |
| Sinha et al. 2010 | PC | TBM | ★★★☆ (3/4) | ★☆ (1/2) | ★★★ (3/3) | 7/9 | Low |
| Hamade et al. 2010 | RC | TBM Treatment | ★★★☆ (3/4) | ★☆ (1/2) | ★★☆ (2/3) | 6/9 | Moderate |
| Amitava et al. 2001 | PC | TBM [PED] | ★★★☆ (3/4) | ☆☆ (0/2) | ★★★ (3/3) | 6/9 | Moderate |
| Lamba et al. 1986 | RC | TBM [PED] | ★★★☆ (3/4) | ☆☆ (0/2) | ★☆☆ (1/3) | 4/9 | Moderate |
| Kalra et al. 1985 | RC | TBM [PED] | ★★★☆ (3/4) | ☆☆ (0/2) | ★☆☆ (1/3) | 4/9 | Moderate |
| Ambika et al. 2022 | RC | EON vs TB-ON | ★★★☆ (3/4) | ★☆ (1/2) | ★★☆ (2/3) | 6/9 | Moderate |
| Davis et al. 2012 | RC | TB-ON | ★★★☆ (3/4) | ☆☆ (0/2) | ★★☆ (2/3) | 5/9 | Moderate |
| **Summary** | **—** | **—** | **Mean: 3.2/4** | **Mean: 0.8/2** | **Mean: 2.2/3** | **Mean: 6.1/9** | **Low: 6 (35%); Moderate: 11 (65%); High: 0 (0%)** |

***Abbreviations:*** *C1, comparability for main confounding factor; C2, comparability for additional factors; CKD, chronic kidney disease; EON, ethambutol optic neuropathy; NOS, Newcastle-Ottawa Scale; O1, assessment of outcome; O2, follow-up length adequacy; O3, adequacy of cohort follow-up; ONP, optic nerve palsy; PB-CC, population-based case-control; PBC, population-based cohort; PC, prospective cohort; PED, pediatric; RC, retrospective cohort; S1, representativeness of exposed cohort; S2, selection of non-exposed cohort; S3, ascertainment of exposure; S4, demonstration outcome not present at start; TB-ON, tuberculous optic neuropathy; TBM, tuberculous meningitis; ★, criterion met; ☆, criterion not met.*
