# Supplementary material for: Neuro-ophthalmic complications of tuberculosis and its treatment: a systematic review and meta-analysis
Source: Front Ophthalmol (Lausanne). 2026 May 29;6:1818640. doi: 10.3389/fopht.2026.1818640 (PMC13259741; doi:10.3389/fopht.2026.1818640)
Supplement: Supplementary file 6 [file Table2.docx]

**Supplementary Table 2:** Sensitivity and Subgroup Analyses for Pooled Estimates.

| **Analysis** | **Studies (k)** | **N** | **Events** | **Pooled Estimate % (95% CI)** | **I² (%)** | **Comparison/Notes** |
| --- | --- | --- | --- | --- | --- | --- |
| Overall Pooled EON Incidence | 5 | 138,863 | 3,606 | 1.54 (0.81–2.49) | 98.2 | Reference estimate |
| Excluding Kim et al. 2024 | 4 | 21,554 | 326 | 1.26 (0.61–2.13) | 95.1 | Leave-one-out |
| Excluding Chaitanuwong et al. 2023 | 4 | 134,722 | 3,586 | 1.91 (1.25–2.70) | 96.5 |  |
| Excluding Chen et al. 2015 | 4 | 134,060 | 3,544 | 1.60 (0.77–2.71) | 98.3 |  |
| Excluding Chen et al. 2012 | 4 | 127,110 | 3,375 | 1.43 (0.46–2.92) | 98.5 |  |
| Excluding Lee et al. 2008 | 4 | 138,006 | 3,593 | 1.53 (0.74–2.60) | 98.6 |  |
| Subgroup: Population-based | 2 | 129,062 | 3,511 | 2.38 (1.63–3.26) | 96.8 | PBC + PB-CC designs |
| Subgroup: Hospital-based | 3 | 9,801 | 95 | 1.03 (0.45–1.85) | 90.2 | RC designs only |
| Subgroup: Pre-2015 studies | 2 | 12,610 | 244 | 1.94 (1.71–2.19) | — | Era subgroup |
| Subgroup: 2015+ studies | 3 | 126,253 | 3,362 | 1.38 (0.32–3.18) | 99.0 |  |
| TBM Mortality: Adult | 3 | 837 | 54 | 8.2 (1.0–21.3) | 96.2 | Population subgroup |
| TBM Mortality: Pediatric | 2 | 148 | 45 | 30.7 (23.5–38.3) | — |  |
| Influence: Kim et al. 2024 | — | 117,309 | 3,280 | 2.80 | — | Weight: 84.5% (High) |
| Influence: Chaitanuwong et al. 2023 | — | 4,141 | 20 | 0.48 | — | Weight: 3.0% (Low) |
| Influence: Chen et al. 2015 | — | 4,803 | 62 | 1.29 | — | Weight: 3.5% (Low) |
| Influence: Chen et al. 2012 | — | 11,753 | 231 | 1.97 | — | Weight: 8.5% (Low) |
| Influence: Lee et al. 2008 | — | 857 | 13 | 1.52 | — | Weight: 0.6% (Low) |

***Abbreviations:*** *CI, confidence interval; EON, ethambutol optic neuropathy; I², heterogeneity statistic; k, number of studies; N, number of patients; PB-CC, population-based case-control; PBC, population-based cohort; RC, retrospective cohort; TBM, tuberculous meningitis.*
