# Supplementary material for: Neuro-ophthalmic complications of tuberculosis and its treatment: a systematic review and meta-analysis
Source: Front Ophthalmol (Lausanne). 2026 May 29;6:1818640. doi: 10.3389/fopht.2026.1818640 (PMC13259741; doi:10.3389/fopht.2026.1818640)
Supplement: Supplementary file 7 [file Table3.docx]

**Supplementary Table 3:** Differential Diagnosis Features: Ethambutol Optic Neuropathy vs Tuberculous Optic Neuropathy.

| **Feature** | **EON (Ambika 2022)** | **TB-ON (Ambika 2022)** | **TB-ON (Davis 2012)** | **Discriminative Value** | **P-value** |
| --- | --- | --- | --- | --- | --- |
| Laterality: Bilateral | 33/35 (94.3%) | 14/37 (37.8%) | 32/62 (51.6%) | Strong: EON predominantly bilateral | <0.001 |
| Laterality: Unilateral | 2/35 (5.7%) | 23/37 (62.2%) | 30/62 (48.4%) | Strong: TB-ON often unilateral | <0.001 |
| Pain on eye movement | 0/35 (0%) | 15/37 (40.5%) | NR | Strong: Pain suggests TB-ON | <0.001 |
| Disc pallor (temporal/diffuse) | 28/35 (80.0%) | 8/37 (21.6%) | 18/62 (29.0%) | Strong: Pallor favors EON | <0.001 |
| Disc edema/hyperemia | 4/35 (11.4%) | 22/37 (59.5%) | 38/62 (61.3%) | Strong: Edema favors TB-ON | <0.001 |
| Normal disc appearance | 3/35 (8.6%) | 7/37 (18.9%) | 6/62 (9.7%) | Weak: Similar rates | 0.21 |
| Red-green dyschromatopsia | 30/35 (85.7%) | 12/37 (32.4%) | NR | Strong: Acquired RG defect suggests EON | <0.001 |
| Central/cecocentral scotoma | 31/35 (88.6%) | 20/37 (54.1%) | 35/62 (56.5%) | Moderate: More common in EON | 0.002 |
| Mean age (years) | 52.3 ± 14.2 | 34.7 ± 12.8 | 38.2 ± 11.5 | Strong: EON in older patients | <0.001 |
| EMB exposure history | 35/35 (100%) | 22/37 (59.5%) | 40/62 (64.5%) | Essential: Required for EON Dx | <0.001 |
| Response to EMB cessation | 22/35 (62.9%) | N/A | N/A | Diagnostic: Improvement confirms EON | — |
| Response to steroids | Minimal | 28/37 (75.7%) | 45/62 (72.6%) | Strong: Steroid response suggests TB-ON | <0.001 |
| Associated CNS tuberculoma | 0/35 (0%) | 12/37 (32.4%) | 15/62 (24.2%) | Strong: Tuberculoma suggests TB-ON | <0.001 |
| Recovery rate (any improvement) | 22/35 (62.9%) | 28/37 (75.7%) | 42/62 (67.7%) | Weak: Similar with treatment | 0.24 |

***Abbreviations:*** *CI, confidence interval; CNS, central nervous system; Dx, diagnosis; EMB, ethambutol; EON, ethambutol optic neuropathy; N/A, not applicable; NR, not reported; RG, red-green; TB-ON, tuberculous optic neuropathy.*
