# Supplementary material for: Neuro-ophthalmic complications of tuberculosis and its treatment: a systematic review and meta-analysis
Source: Front Ophthalmol (Lausanne). 2026 May 29;6:1818640. doi: 10.3389/fopht.2026.1818640 (PMC13259741; doi:10.3389/fopht.2026.1818640)
Supplement: Supplementary file 8 [file Table4.docx]

**Supplementary Table 4:** Publication Bias Assessment and GRADE Evidence Profile.

| **Assessment Domain** | **Outcome/Criterion** | **Result** | **Statistical Test** | **Interpretation** |
| --- | --- | --- | --- | --- |
| Egger regression intercept | EON Incidence | Intercept = −8.476 | t-test p < 0.001 | Significant asymmetry |
| Begg rank correlation |  | τ = −0.600 | p = 0.233 | No significant asymmetry |
| Funnel plot inspection |  | Visual assessment | k = 5 studies | Limited studies for robust assessment |
| Trim-and-fill estimate |  | Not performed | k < 10 | Insufficient studies |
| GRADE: Risk of bias |  | Not serious | 2/5 low RoB | No downgrade |
| GRADE: Inconsistency |  | Serious | I² = 98.2% | Downgrade 1 level |
| GRADE: Indirectness |  | Not serious | Direct evidence | No downgrade |
| GRADE: Imprecision |  | Not serious | N = 138,863 | No downgrade |
| GRADE: Publication bias |  | Undetected | Begg p = 0.233 | No downgrade |
| GRADE: Overall certainty |  | MODERATE | ⊕⊕⊕⊖ | Downgraded for inconsistency |
| GRADE: Risk of bias | EON Risk Factors | Not serious | Chen 2012: Low RoB | No downgrade |
| GRADE: Inconsistency |  | Not serious | Consistent direction | No downgrade |
| GRADE: Indirectness |  | Serious | Single population | Downgrade 1 level |
| GRADE: Imprecision |  | Not serious | N = 11,753 | No downgrade |
| GRADE: Overall certainty |  | MODERATE | ⊕⊕⊕⊖ | Downgraded for indirectness |
| GRADE: Risk of bias | TBM Manifestations | Serious | 1/6 low RoB | Downgrade 1 level |
| GRADE: Inconsistency |  | Serious | I² = 92–99% | Downgrade 1 level |
| GRADE: Indirectness |  | Not serious | Direct evidence | No downgrade |
| GRADE: Imprecision |  | Serious | Wide CIs | Downgrade 1 level |
| GRADE: Overall certainty |  | VERY LOW | ⊕⊖⊖⊖ | Downgraded 3 levels |
| GRADE: Risk of bias | Subclinical Biomarkers | Not serious | 2/2 low RoB | No downgrade |
| GRADE: Inconsistency |  | Not serious | Consistent findings | No downgrade |
| GRADE: Indirectness |  | Serious | Surrogate outcomes | Downgrade 1 level |
| GRADE: Imprecision |  | Not serious | Narrow CIs | No downgrade |
| GRADE: Overall certainty |  | MODERATE | ⊕⊕⊕⊖ | Downgraded for indirectness |
| GRADE: Risk of bias | Visual Recovery | Serious | Variable RoB | Downgrade 1 level |
| GRADE: Inconsistency |  | Not serious | I² = 30.9% | No downgrade |
| GRADE: Indirectness |  | Not serious | Direct evidence | No downgrade |
| GRADE: Imprecision |  | Serious | N = 116 | Downgrade 1 level |
| GRADE: Overall certainty |  | LOW | ⊕⊕⊖⊖ | Downgraded 2 levels |

***Abbreviations:*** *CI, confidence interval; EON, ethambutol optic neuropathy; GRADE, Grading of Recommendations Assessment Development and Evaluation; I², heterogeneity statistic; k, number of studies; N, number of patients; RoB, risk of bias; TBM, tuberculous meningitis; τ, Kendall's tau; ⊕, criterion met; ⊖, criterion not met.*
