# Supplementary material for: Neuro-ophthalmic complications of tuberculosis and its treatment: a systematic review and meta-analysis
Source: Front Ophthalmol (Lausanne). 2026 May 29;6:1818640. doi: 10.3389/fopht.2026.1818640 (PMC13259741; doi:10.3389/fopht.2026.1818640)
Supplement: Supplementary file 9 [file Table5.docx]

**Supplementary Table 5:** Choroidal Tubercles, MRC Grade Distribution, and Supplementary Clinical Data.

| **Data Category** | **Study** | **Parameter** | **N** | **Value** | **95% CI** | **Clinical Significance** |
| --- | --- | --- | --- | --- | --- | --- |
| Choroidal Tubercles | Amitava et al. 2001 | Prevalence | 100 | 12/100 (12.0%) | 7.0–20.0% | Pathognomonic for TB when present |
|  | Amitava et al. 2001 | Association with mortality | 100 | 5/12 (41.7%) vs 25/88 (28.4%) | OR 1.8 (0.5–6.2) | Trend toward higher mortality |
|  | Kalra et al. 1985 | Prevalence | 50 | 4/50 (8.0%) | 3.2–18.8% | Less common in pediatric TBM |
| MRC Grade Distribution | Fei et al. 2024 | Grade I | 250 | 136 (54.4%) | 48.2–60.5% | Majority early-stage |
|  | Fei et al. 2024 | Grade II | 250 | 83 (33.2%) | 27.6–39.3% | Intermediate stage |
|  | Fei et al. 2024 | Grade III | 250 | 31 (12.4%) | 8.8–17.2% | Severe stage |
|  | Li et al. 2019 | Grade I | 486 | 234 (48.1%) | 43.7–52.6% | Largest cohort |
|  | Li et al. 2019 | Grade II | 486 | 133 (27.4%) | 23.5–31.6% | Intermediate stage |
|  | Li et al. 2019 | Grade III | 486 | 119 (24.5%) | 20.8–28.5% | Higher severe proportion |
|  | Sinha et al. 2010 | Grade I | 101 | 21 (20.8%) | 13.9–30.0% | Referral center bias |
|  | Sinha et al. 2010 | Grade II | 101 | 39 (38.6%) | 29.6–48.5% | Intermediate stage |
|  | Sinha et al. 2010 | Grade III | 101 | 41 (40.6%) | 31.3–50.5% | High severe proportion |
| EMB Cumulative Dose | Chen et al. 2015 | Mean cumulative dose | 62 | 2,820 ± 2,157 mg/kg | — | EON cohort total exposure |
|  | Chen et al. 2015 | Daily dose (EON cohort) | 62 | 16.06 ± 4.3 mg/kg/day | — | Higher than recommended |
|  | Lee et al. 2008 | Daily dose (EON cohort) | 13 | 17.85 ± 2.2 mg/kg/day | — | Exceeds 15 mg/kg threshold |
| Time-to-Event | Kim et al. 2024 | 1-year cumulative incidence | 117,309 | 2.1% | — | Kaplan-Meier estimate |
|  | Kim et al. 2024 | 2-year cumulative incidence | 117,309 | 2.8% | — | Plateau effect observed |
|  | Lee et al. 2008 | Mean time to EON onset | 13 | 5.8 months | — | Peak risk period 2–6 mo |
|  | Kim et al. 2016 | Subclinical onset | 31 | 2–5 months | — | Earlier than clinical |
| Recovery by Severity | Chen et al. 2015 | Improved group age | 31 | 74.8 ± 14 years | — | Younger = better prognosis |
|  | Chen et al. 2015 | Non-improved group age | 31 | 78.6 ± 12 years | — | p = 0.095 (trend) |
|  | Ambika et al. 2022 | Overall recovery | 35 | 22/35 (62.9%) | 46.3–76.8% | Tertiary center |
|  | Lee et al. 2008 | Recovery (severe cases) | 13 | 4/13 (30.7%) | 12.7–57.6% | Poor in advanced EON |

***Abbreviations:*** *CI, confidence interval; EMB, ethambutol; EON, ethambutol optic neuropathy; mo, months; MRC, Medical Research Council; N, number; OR, odds ratio; TB, tuberculosis; TBM, tuberculous meningitis.*
